# Sagittal balance is more than just alignment: why PJK remains an unresolved problem

**DOI:** 10.1186/s13013-016-0064-0

**Published:** 2016-01-22

**Authors:** Steven D. Glassman, Mark P. Coseo, Leah Y. Carreon

**Affiliations:** Norton Leatherman Spine Center, 210 East Gray Street, Suite 900, Louisville, Kentucky 40202 USA

**Keywords:** Adult spinal deformity, Adult scoliosis, Proximal junctional kyphosis, PJK

## Abstract

**Background:**

The durability of adult spinal deformity surgery remains problematic. Revision rates above 20 % have been reported, with a range of causes including wound infection, nonunion and adjacent level pathology. While some of these complications have been amenable to changes in patient selection or surgical technique, Proximal Junctional Kyphosis (PJK) remains an unresolved challenge. This study examines the contributions of non-mechanical factors to the incidence of postoperative sagittal imbalance and PJK after adult deformity surgery.

**Methods:**

We reviewed a consecutive series of adult spinal deformity patients who required revision for PJK from 2013 to 2015 and examined in their medical records in detail.

**Results:**

Neurologic disorders were identified in 22 (76 %) of the 29 PJK cases reviewed in this series. Neurologic disorders included Parkinson’s disease (1), prior stroke (5), metabolic encephalopathy (2), seizure disorder (1), cervical myelopathy (7), thoracic myelopathy (1), diabetic neuropathy (5) and other neuropathy (4). Other potential comorbidities affecting standing balance included untreated cataracts (9), glaucoma (1) and polymyositis (1). Eight patients were documented to have frequent falls, with twelve cases having a fall right before symptoms related to the PJK were noted.

**Conclusion:**

PJK is an important contributing factor to the substantial and unsustainable rate of revision surgery following adult deformity correction. Multiple efforts to avoid PJK via alterations in surgical technique have been largely unsuccessful. This study suggests that non-mechanical neuromuscular co-morbidities play an important role in post-operative sagittal imbalance and PJK. Recognizing the multi-factorial etiology of PJK may lead to more successful strategies to avoid PJK and improve surgical outcomes.

## Background

Surgical treatment of adult spinal deformity has progressed substantially over the past ten years. There have been significant advances in decision-making, medical management and surgical technique [1, 2]. These improvements in evaluation and treatment have broadened the applicability of adult deformity surgery and lead to more reproducible clinical benefit based upon health related quality of life (HRQOL) scores [3, 4].

Despite these positive developments, the durability of adult spinal deformity surgery remains problematic. Revision rates above 20 % have been reported, with a range of causes including wound infection, nonunion and adjacent level pathology [5–7]. While some of these complications have been amenable to changes in patient selection or surgical technique, Proximal Junctional Kyphosis (PJK) remains an unresolved challenge.

The initial description of PJK in the pediatric literature was an increased sagittal angulation, without structural failure, at the upper aspect of a fusion construct [8]. At present, the term is applied much more widely to describe any failure or loss of alignment above an instrumented segment [9, 10]. This may result from adjacent level compression fracture, spondylolisthesis or fixation failure [6–10]. In general, this has been viewed as a consequence of poor bone quality, over-aggressive deformity correction or inadequate fixation.

PJK has been the focus of intense scrutiny, with multiple studies proposing mechanical solutions including adaptations for osteoporotic bone and in particular specific sagittal alignment targets [11, 12]. Unfortunately, none of these mechanical solutions have effectively decreased the rate of PJK. The role of this study is to examine the contributions of non-mechanical factors to the incidence of postoperative sagittal imbalance and PJK after adult deformity surgery.

## Methods

After receiving Institutional Review Board Approval, we reviewed a consecutive series of adult spinal deformity patients who required revision for PJK from 2013 to 2015 and examined in their medical records in detail. Standard demographic data including age, gender, smoking status, height and weight were collected. Indications for the index surgery, specifics of the index surgery including upper instrumented vertebra fixation, time to PJK diagnosis, time to PJK surgery, mode of failure. Medical records were extensively evaluated for preoperative comorbidities; specifically for preoperative neurologic disorders and other pathologies that may affect standing balance.

## Results

From 2012 to 2014, 245 patients underwent surgical correction of their adult spinal deformity at our institution. A true incidence of PJK will be difficult to determine as (1) some patients presenting at our institution with PJK had their index surgery performed elsewhere and (2) some of the patients who had their index surgery at our institution could have developed PJK and had surgery elsewhere.

Twenty-nine cases of PJK requiring revision were identified (Table 1). Of these 9 (31 %) were males and 10 (34 %) were smokers. Mean age was 64.4 years. Mean BMI was 29. kg/m^2^. Neurologic disorders were identified in 22 (76 %) of the PJK cases reviewed in this series. Neurologic disorders included Parkinson’s disease (1), prior stroke (5), metabolic encephalopathy (2), seizure disorder (1), cervical myelopathy (7), thoracic myelopathy (1), diabetic neuropathy (5) and other neuropathy (4). Other potential comorbidities affecting standing balance included untreated cataracts (9), glaucoma (1) and polymyositis (1) (Table 2). Eight patients were documented to have frequent falls, with twelve cases having a fall right before symptoms related to the PJK were noted. Seventeen cases used an assistive device such as a cane, crutches or a walker and required a wheelchair. One patient had 5 co-morbid conditions affecting standing balance, two had 4 co-morbid conditions, four had 3 co-morbid conditions, nine had 2 co-morbid conditions and ten had only one co-morbid condition (Table 3).

**Table 1:** Summary of cases

Case. No.	Age/Sex	Smoker	BMI	Indication for Index Surgery	Index Surgery	UIV Fixation	Time to PJK diagnosis	Mode of Failure	PJK surgery	Fall prior	Assistive devise	CCMI	Other co-morbidities
1	68/F	Yes	40.9	Kyphoscoliosis	PSF T10 to Pelvis, TLIF L3-L4	bilateral pedicle screws	8 months	Fracture of T9-T10 with cord compression	T9-T10 laminectomy, extension of fusion T4-T11	No	No	11	None
2	64/M	Yes	19.7	Stenosis	PSF L3 to L5	bilateral pedicle screws	18 months	Fracture of L3	PSO L3, PSF T11 to pelvis	Yes	Cane	11	CVA, Loss of reflexes below knee
3	58/M	No	33.9	Multilevel stenosis	PSF L3 to Pelvis	bilateral pedicle screws	17 months	Fracture of L3	AIF L5-S1, Ponte osteotomies, PSF T10 to pelvis	Yes	No	4	CSM post ACDF
4	63/F	No	25.9	Multilevel stenosis	PSF L2 to L5	bilateral pedicle screws	21 months	Compression of L2 with complete loss of L1-L2 interspace	Extension to T10	No	Wheelchair	10	CVA, Cauda equina requiring emergent decompression, Diabetic neuropathy
5	65/F	Yes	34.9		ASF L4-S1, PSF T10 to Pelvis	bilateral pedicle screws	11 months	Compression Fracture T11	Extension of fusion to T3	Yes	Walker	9	Diabetic neuropathy, Frequent falls, post bilateral TKA, ORIF L ankle
6	70/F	No	25.7	Kyphoscoliosis	ASF, PSF T10 to Pelvis	bilateral pedicle screws	12 months	Compression Fracture T9	Extension of fusion to T3	No	Cane	7	Cataracts
							1 month after 1st PJK	Pull out of claw construct fracturing T4 to T8 laminae	Extension of Fusion T2 to T12				
7	52/F	Yes	25.6	Degenerative scoliosis, stenosis	PSF, L2 to sacrum	bilateral pedicle screws	64 months	Kyphosis at L1-L2 impingement of screws into disc space	TLIF L1-L2, PSF L1-L2	Yes	Crutches	8	Diabetic neuropathy
8	64/M	No	31.0	Flatback S/P L3-L5 PSF	ASF L5-S1, PSF T9 to Sacrum	bilateral pedicle screws	18 months	T8-T9 Listhesis	Extension of fusion to T2	Yes	Walker	8	CVA, Neuropathy, Cataracts (removed), CSM post laminectomy, Frequent falls, post THA dislocation
9	57/F	No	30.5	Flatback S/P L3-L5 laminectomies	PSF T11 to Pelvis	bilateral pedicle screws	25 months	Compression Fracture T9 - T10	Extension of fusion to T3	No	No	9	TIAs, Diabetic neuropathy, Cataracts, Frequent falls, post bilateral TKA, multiple foot surgeries
10	60/M	Yes	19.3	Kyphoscoliosis	ASF L4-S1, PSF T10 to Pelvis	bilateral pedicle screws	82 months	Fracture of T9, T8-T9 spondylolisthesis	PSF T4 to T12	Yes	Walker	9	CVA, Sensory neuropathy, Glaucoma, Frequent falls, post multiple revisions of bilateral TKA
							14 months after 1st PJK	Pull out of claw construct fracturing T3 lamina	Extension of Fusion T1 to T10				
11	58/M	No	34.7	Degenerative scoliosis, stenosis	PSF T10 to Pelvis, TLIF L5-S1	bilateral pedicle screws	1 month	T9-T10 Listhesis	PSF T4 to T10	Yes	No	6	DTs, Neuropathy, Frequent falls, alcoholic, had DTs after index surgery
12	75/F	No	29.0	Degenerative scoliosis S/P L2-L3 PDSF	PSF T10 to Pelvis, TLIF L5-S1	bilateral pedicle screws	27 months	Screw pull out	Extension to T4	Yes	No	5	None
13	62/F	Yes	30.0	Flatback deformity S/P L2-LS1 PDSF	PSF L2 to S1	bilateral pedicle screws	12 months	Fracture L1	Extension to T10	No	Cane	6	Tremors, Multiple foot surgeries
							38 months after 1st PJK	T9-T10 fracture with erosion of screws into disc	Removal of instrumentation, PSF T4 to L2				
14	69/F	Yes	26.5	Adjacent segment degeneration S/P L3 to L5 PSF	Extension of fusion L1 to S1	bilateral pedicle screws	1 month	L1-L2 listhesis	Extension from T10 to S1	No	No	7	None
15	57/F	No	40.8	Degenerative scoliosis, stenosis	PSF L2 to S1	bilateral pedicle screws	92 months	L1-L2 listhesis	Extension from T10 to S1	No	Cane	5	Parkinson's disease
16	63/F	No	23.2	Adjacent segment stenosis S/P L1 to S1 PSDF	PSF T9 to L3	bilateral pedicle screws	7 months	Posterior lysis of T9 and T10	Removal of instrumentation, PSF T4 to L3	Yes	Cane	8	Cataract, CSM post C3 to T1 ACDF
17	73/F	No	36.4	Scoliosis	PSF T6 to Sacrum	bilateral hooks	80 months	Fracture T6	Removal of instrumentation, PSF T3 to L1	No	No	9	Cataract
18	61/M	Yes	32.5	Scoliosis	PSF T8 to Sacrum	bilateral pedicle screws	11 months	Fracture T8	Removal of instrumentation, PSF T4 to Pelvis	No	Walker	7	Polymyositis
19	72/M	No	36.8	Scoliosis	PSF T11 to L3	bilateral pedicle screws	23 months	T10-T11 listhesis	Removal of instrumentation, PSF T8 to T11	No	Walker	6	CSM post laminoplasty, Frequent falls
							41 months after 1st PJK	T7-T8 listhesis	Removal of instrumentation T9-L1, PSF T2 to T9				
20	78/F	No	39.9	Scoliosis	PSF L1 to S1	bilateral pedicle screws	14 months	Fracture T12	Extension of Fusion to T8	No	No	7	Metabolic encephalopathy, Cataract
21	71/F	Yes	26.5	Degenerative Scoliosis	PSF L2-L3	bilateral pedicle screws	45 months	L1-L2 collapse and localized scoliosis	Extension to T10	No	Cane	10	Cataract (removed), Cervical osteomyelitis with cord compromise
22	75/F	No	30.4	Scoliosis	PSF T4 to Pelvis	bilateral hooks	2 months	Hook pull-out with T4-T6 laminar fractures	Extension to T2	No	No	10	Cataract
23	69/M	No	27.4	Post-laminectomy instability	ASF L3 to S1, PSF L2 to S1	bilateral pedicle screws	1 month	Compression Fracture of L2 with screw pullout	Removal of instrumentation, PSF T10 to L1	No	No	6	Diabetic neuropathy
24	55/F	No	38.0	Adjacent segment stenosis S/P L2 to S1 PSDF	PSF T10 to Pelvis, TLIF L2-L3, L5-S1	bilateral pedicle screws	9 months	Compression Fracture T10	Extension to T3	Yes	No	8	CSM post ACDF
25	70/F	No	35.5	Stenosis	PDSF L2-L5	bilateral pedicle screws	10 months	Compression of L2	PSF T10 to Pelvis	Yes	Cane	7	Metabolic encephalopathy, Cataract, Frequent falls
							5 months after 1st PJK	Compression Fracture T9	Extension to T2				
26	62/F	No	21.4	Adjacent segment stenosis S/P L3 to L5 PSDF	ASF L2 to L5, Extension of fusion to T10	bilateral hooks	3 months	Fracture T10	Extension to T2	No	Cane	No	CSM post ACDF, Neuropathy, Frequent falls
27	73/F	No	20.6	Scoliosis	PSF T10 to Pelvis	bilateral pedicle screws	4 months	T10 compression fracture	PSF T7 to T12	Yes	Walker	Yes	Mild cognitive impairment, Benign thoracic tumor S/P excision
28	65/F	No	21.1	Scoliosis	PSF T11 to S1	bilateral pedicle screws	22 months	T10-T11 listhesis, nonunion L5-S1	AIF L3 to S1, PSF T10 to Pelvis	No	No	No	Seizures, Eye surgery
29	33/M	Yes	26.7	Scoliosis	PSF L1 TO L4	bilateral pedicle screws	22 months	Compression of T12	Removal of instrumentation, PSF T10 to Pelvis	No	Cane	No	Chronic dropfoot

*PSDF* posterior spinal decompression and fusion, *PSF* posterior spinal fusion, *ASF* anterior spinal fusion, *TLIF* transforaminal lumbar interbody fusion, *CVA* cerebrovascular accident, *CSM* cervical spondylotic myelopathy, *ACDF* anterior cervical discectomy and fusion, *TKA* total knee arthroplasty, *ORIF* open reduction internal fixation, *THA* total hip arthroplasty, *DT* delirium tremens

**Table 2:** Frequency of co-morbid conditions that can affect balance

Co-morbid condition	Frequency
Prior stroke	5
Metabolic encephalopathy	2
Parkinson's disease	1
Seizures	1
Polymyositis	1
Diabetic Neuropathy	5
Neuropathy	4
Cataract	9
Glaucoma	1
Myelopathy	8
Frequent falls	8

**Table 3:** Number of co-morbid conditions that can affect balance

	Frequency
None	3
One	10
Two	9
Three	4
Four	2
Five	1

## Discussion

Proximal Junctional Kyphosis was first identified in 1999 [8], and was initially described as a radiographic finding with limited clinical relevance [13, 14]. This sanguine assessment was short lived, as subsequent reports have documented the frequent need for revision surgery [5, 6] as well as the occurrence of catastrophic failures, termed Proximal Junctional Failure (PJF) [9, 10, 15, 16]. The reported increase in PJK was coincident with several major changes in treatment paradigm. Adult deformity surgery became more common in older patients, and more aggressive correction was undertaken using osteotomies and rigid instrumentation. Studies have highlighted these factors and examined their etiologic role in PJK and PJF [10, 17, 18].

Deformity surgeons clearly recognize PJK and PJF as important challenges, but often regard these complications as mechanical problems for which there should be a straight forward mechanical solutions. As osteoporosis is commonly identified as an etiology of PJK, surgeons have pursued options to offset poor bone quality. Strategies have included prophylactic medical treatment of low bone density, strengthening proximal instrumented and adjacent vertebral levels with cement injection. Other strategies have included decreasing rod rigidity, and softening the transition to unfused levels using hooks rather than screws [11, 19, 20]. Another major focus has been on selection of fusion levels and restoration of sagittal alignment [12, 18, 21, 22]. Studies have advocated both more aggressive and less aggressive deformity correction. Maruo et al. report that restoration of normal sagittal alignment protected against PJK, and that greater than 30-degree increase in lumbar lordosis was a significant risk factor for PJK. [18] As increase in lumbar lordosis is generally the mechanism by which normal sagittal alignment is restored, these observations appear contradictory.

The findings of the present study suggest that our failure to control the rate of PJK may be related in part to the narrow focus on mechanical factors. This study demonstrates that 76 % of patients with PJK after spinal deformity correction have co-morbidities that adversely affect standing balance, regardless of alignment. These include neuromuscular disease, history of cerebral vascular accident, cervical myelopathy and neuropathy. All of these conditions may contribute to an inability to rebalance through unfused segments after deformity correction. This phenomenon is clearly recognized with substantial neurologic impairment such as patients with Parkinson’s disease [23], but has not been clearly defined in those patients with less severe neurologic impairment.

Beyond potential neurogenic causes of standing imbalance, other factors such as visual impairment, vestibular dysfunction and severe muscular deconditioning also impact balance and gait [24, 25]. Visual impairment was noted in 40 % of PJK cases and more than a single potentially relevant co-morbidity was noted in more than 66 % of cases. While these findings do not implicate neuromuscular disease as the direct cause of PJK, they certainly suggest a multi-factorial etiology.

The mechanisms by which these non-mechanical risk factors contribute to PJK are not well defined, and probably do not represent a unique common pathway. In some instances, such as patients with neuropathy or central neurologic deterioration, an impaired feedback loop may limit the ability to compensate appropriately after mechanical realignment. In essence, the patient’s brain does not properly register the “improved alignment” as determined by radiographic assessment. In other cases, lack of appropriate sensory feedback may result in accelerated proximal segment degeneration, akin to the appearance of a Charcot joint. In patients with severe deconditioning, muscular support may be inadequate regardless of mechanical alignment.

It is not completely clear how best to apply these observations in clinical practice. Our case series methodology cannot provide a relative risk assessment for any of the individual co-morbid conditions, and to-date no diagnostic test has been developed to quantify a global risk for post-operative standing imbalance or PJK. It is also unknown as to whether these risks can be modified by pre-operative interventions such as balance training, in the same way that treatment of osteoporosis is thought to reduce the risk of post-operative vertebral fracture or screw pull-out.

Weaknesses of this study include firstly the case series methodology. As some of the patients had their index procedure elsewhere, we do not have an accurate denominator to assess the incidence of PJK in the primary cohort. This series is also relatively small, so that the relative risk of the various co-morbidities cannot be effectively compared. Despite these weaknesses, this study clearly supports the role of concomitant neuromuscular disease in the development of post-op standing imbalance and PJK. The data does not provide a specific threshold at which surgery should be withheld, but certainly emphasizes the importance of including an assessment of associated neuromuscular disease in pre-operative planning and shared decision-making.

Spine surgeons have devoted a great deal of time and effort to defining optimal sagittal alignment, but sagittal balance is more than just alignment. Dubousset outlined the many interactive systems that contribute to ambulation and stated, “good alignment is preferable in order to obtain a good balance, but it is not sufficient” [26]. Understanding and avoiding PJK requires that we move beyond the one-dimensional view that finding an ideal sagittal alignment, softening the transition at the proximal aspect of the instrumented segment, or improving the adjacent bone strength will solve the problem of PJK. Thinking about PJK more broadly is a step in the right direction.

## Conclusions

PJK is an important contributing factor to the substantial and unsustainable rate of revision surgery following adult deformity correction. Multiple efforts to avoid PJK via alterations in surgical technique have been largely unsuccessful. This study suggests that non-mechanical neuromuscular co-morbidities play an important role in post-operative sagittal imbalance and PJK. Recognizing the multi-factorial etiology of PJK may lead to more successful strategies to avoid PJK and improve surgical outcomes.
